# Living With HIV Through the COVID-19 Pandemic: Impacts on Older Adults in New York City

**DOI:** 10.1093/geroni/igab046.846

**Published:** 2021-12-17

**Authors:** Annie Nguyen, Stephan Karpiak

**Affiliations:** 1 University of Southern California, Alhambra, California, United States; 2 GHMC, New York, New York, United States

## Abstract

New York City was among the first to institute physical distancing and shutdowns to curb community spread of COVID-19. The pandemic has amplified issues related to isolation. We investigated the challenges created by the pandemic older adults living with HIV in NYC. 137 participants were recruited Sept-Nov 2020 from the oldest ASO in NYC, to complete surveys. Demographics: mean age=60.4; 58.3% men; 43.1% black/AA, 24.1% white; 48.9% gay, 30.7% straight; mean years living with HIV= 23.0, 92.6% reported undetectable viral loads. About one-third experienced hunger/food insecurity during the pandemic and 48.2% said they were not getting enough financial support from usual sources. Some (43.3%) reported skipping doses of HIV medications and 69.8% felt more isolated compared to before the pandemic. Those who lived alone (77.4% of total) were significantly more likely to report feeling depressed, follow media coverage on COVID-19, skip HIV medications, and experience changes in sleep patterns.

